# Perfusion assessment by fluorescence time curves in esophagectomy with gastric conduit reconstruction: a prospective clinical study

**DOI:** 10.1007/s00464-023-10107-9

**Published:** 2023-05-19

**Authors:** J. J. Joosten, M. D. Slooter, R. M. van den Elzen, P. R. Bloemen, S. S. Gisbertz, W. J. Eshuis, F. Daams, D. M. de Bruin, M. I. van Berge Henegouwen

**Affiliations:** 1 Department of Surgery, Amsterdam UMC Location University of Amsterdam, Meibergdreef 9, Amsterdam, The Netherlands; 2 Cancer Center Amsterdam, Imaging and Biomarkers, Amsterdam, The Netherlands; 3 Department of Biomedical Engineering, Amsterdam UMC Location University of Amsterdam, Amsterdam, The Netherlands; 4 Department of Surgery, Amsterdam UMC Location Vrije Universiteit Amsterdam, Amsterdam, The Netherlands

**Keywords:** Fluorescence angiography (FA), Fluorescence time curves, Indocyanine green (ICG), Esophagectomy, Anastomotic leakage

## Abstract

**Background:**

Intraoperative perfusion assessment with indocyanine green fluorescence angiography (ICG-FA) may reduce postoperative anastomotic leakage rates after esophagectomy with gastric conduit reconstruction. This study evaluated quantitative parameters derived from fluorescence time curves to determine a threshold for adequate perfusion and predict postoperative anastomotic complications.

**Methods:**

This prospective cohort study included consecutive patients who underwent FA-guided esophagectomy with gastric conduit reconstruction between August 2020 and February 2022. After intravenous bolus injection of 0.05-mg/kg ICG, fluorescence intensity was registered over time by PINPOINT camera (Stryker, USA). Fluorescent angiograms were quantitatively analyzed at a region of interest of 1 cm diameter at the anastomotic site on the conduit using tailor-made software. Extracted fluorescence parameters were both inflow (T_0_, T_max_, F_max_, slope, Time-to-peak) as outflow parameters (T_90%_ and T_80%_). Anastomotic complications including anastomotic leakage (AL) and strictures were documented. Fluorescence parameters in patients with AL were compared to those without AL.

**Results:**

One hundred and three patients (81 male, 65.7 ± 9.9 years) were included, the majority of whom (88%) underwent an Ivor Lewis procedure. AL occurred in 19% of patients (*n* = 20/103). Both time to peak as T_max_ were significantly longer for the AL group in comparison to the non-AL group (39 s vs. 26 s, p = 0.04 and 65 vs. 51 s, *p* = 0.03, respectively). Slope was 1.0 (IQR 0.3–2.5) and 1.7 (IQR 1.0–3.0) for the AL and non-AL group (*p* = 0.11). Outflow was longer in the AL group, although not significantly, T_90%_ 30 versus 15 s, respectively, *p* = 0.20). Univariate analysis indicated that T_max_ might be predictive for AL, although not reaching significance (*p* = 0.10, area under the curve 0.71) and a cut-off value of 97 s was derived, with a specificity of 92%.

**Conclusion:**

This study demonstrated quantitative parameters and identified a fluorescent threshold which could be used for intraoperative decision-making and to identify high-risk patients for anastomotic leakage during esophagectomy with gastric conduit reconstruction.

A significant predictive value remains to be determined in future studies.

Esophagectomy with gastric conduit reconstruction is an essential part of multimodal curative treatment of resectable esophageal cancer [1]. Anastomotic leakage (AL) remains a life-threatening complication with an incidence of 7–30% [2]. The most common risk factors for AL of the esophagogastrostomy are torsion of or tension on the anastomosis, location of anastomosis, surgeon experience, active smoking, and corticosteroid therapy [3, 4]. Another important risk factor is poor blood supply at the anastomotic site. The gastric conduit is especially at risk as it mainly relies on the right gastro-epiploic artery and right gastric artery for its blood supply [5]. Among these risk factors, only perfusion and anastomotic tension/torsion can be intervened upon intraoperatively [6]. However, intraoperative evaluation of gastrointestinal perfusion is challenging. Studies on this subject are lacking uniformity in approach, reliability, and objectivity. Indocyanine green fluorescence angiography (ICG-FA) is a promising tool to demonstrate adequate perfusion. Although, at this moment, the use of this technique contends with similar shortcomings as previous intraoperative tools with regard to subjectivity and inter-user variability [7]. Possibly partly due to these factors, studies show inconsistent results of the effect of ICG-FA on anastomotic leakage rates [8–11]. 

Ideally, a quantitative threshold for the fluorescence signal will be identified to predict adequate perfusion and postoperative outcomes. In order to establish a threshold, numerous research teams have been searching for quantifiable fluorescence parameters, from relatively simple quantification methods, such as time to fluorescence, to more complex methods, such as fluorescence time curves [12, 13]. However, all of these studies were retrospectively executed or had small sample sizes.

This study evaluates various parameters derived from fluorescence time curves as a quantitative value for ICG-FA and aims to determine a threshold to predict anastomotic complications in patients undergoing esophagectomy with gastric conduit reconstruction.

## Methods

### Study design

In this single-center prospective study, we included consecutive patients that underwent esophagectomy in Amsterdam UMC from August 2020 until February 2022.

Patients were included when they met the following criteria: 18 years or older and esophagectomy with gastric conduit reconstruction (Ivor Lewis or McKeown procedure). Exclusion criteria were no informed consent, robot-assisted procedures (due to different instrument ports) or allergy to ICG, iodide, or sodium iodide. FA data were recorded in a prospectively maintained database. Patient data were extracted from a prospectively maintained database. The Institutional Review Board of the Amsterdam UMC location University of Amsterdam approved the study protocol and confirmed that the Medical Research lnvolving Human Subjects Act (WMO) did not apply.

### Surgical procedure

Before surgery, patients standardly received neoadjuvant treatment, usually consisting of chemoradiotherapy or perioperative chemotherapy [14, 15]. Based on the primary tumor location and the radiation field, patients underwent either an Ivor Lewis or a McKeown procedure, as previously described [16]. In brief, after mobilization of the esophagus and intrathoracic and abdominal lymphadenectomy, ligation of the left gastric artery, right gastric artery at the angulus of the stomach, the left gastro-epiploic artery, and the short gastric vessels was performed. A 3–4-cm-wide gastric tube was constructed. In Ivor Lewis esophagectomy, an intrathoracic anastomosis was created with a stapled anastomosis. The anastomosis was covered by an omental wrap and mediastinal pleural flap [17].

During the abdominal phase of the McKeown procedure, the gastric conduit was constructed through a small upper abdominal midline laparotomy when a minimally invasive approach was followed. Consequently, a left cervical incision was made, the gastric conduit was brought up to the cervical region through the prevertebral route, and a hand-sewn or cervical anastomosis was created and wrapped with omentum. A pyloromyotomy or pyloroplasty was not performed at our center.

### Standardized fluorescence assessment

ICG-FA was performed both before and after creation of the anastomosis, after the gastric conduit was brought up into the thorax (Ivor Lewis procedure) or exteriorly through the abdominal incision (McKeown procedure). Before ICG-FA, the planned anastomotic site of the gastric conduit was determined by visual inspection and measuring the needed gastric conduit length which was marked.

During the McKeown procedure, an estimation was made on the predicted gastric conduit length; this point was marked during assessment. Subsequently, the camera was fixed in a laparoscopic holder 9 cm from the planned anastomotic site.

All surrounding light was turned off. ICG-FA was performed after administration of ICG (0.05 mg/kg/bolus) through a peripheral infusion cannula and FA images were captured for 200 s. Post-anastomotic assessment was performed, in which the laparoscopic camera was fixed 6 cm from the anastomosis in a laparoscopic holder and FA was captured for 200 s. This distance was chosen for a more optimal view of the gastric conduit after anastomosis.

The laparoscopic PINPOINT camera (Stryker, Kalamazoo, MI, USA) was used to detect ICG. Based on the subjective interpretation of the fluorescence enhancement ICG-FA assessment, the surgeon was allowed to prompt change in surgical management. Change in management included extra mobilization or higher pull up of the gastric conduit or choosing a more proximal anastomotic site with additional resection of the gastric conduit.

### Quantification of fluorescent imaging

**Fig. 1:**
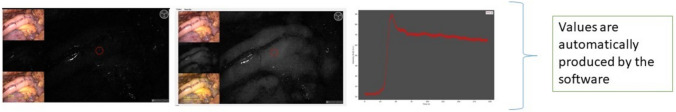
Represents region of interest (ROI) selection and how the fluorescence time curve is produced

**Fig. 2:**
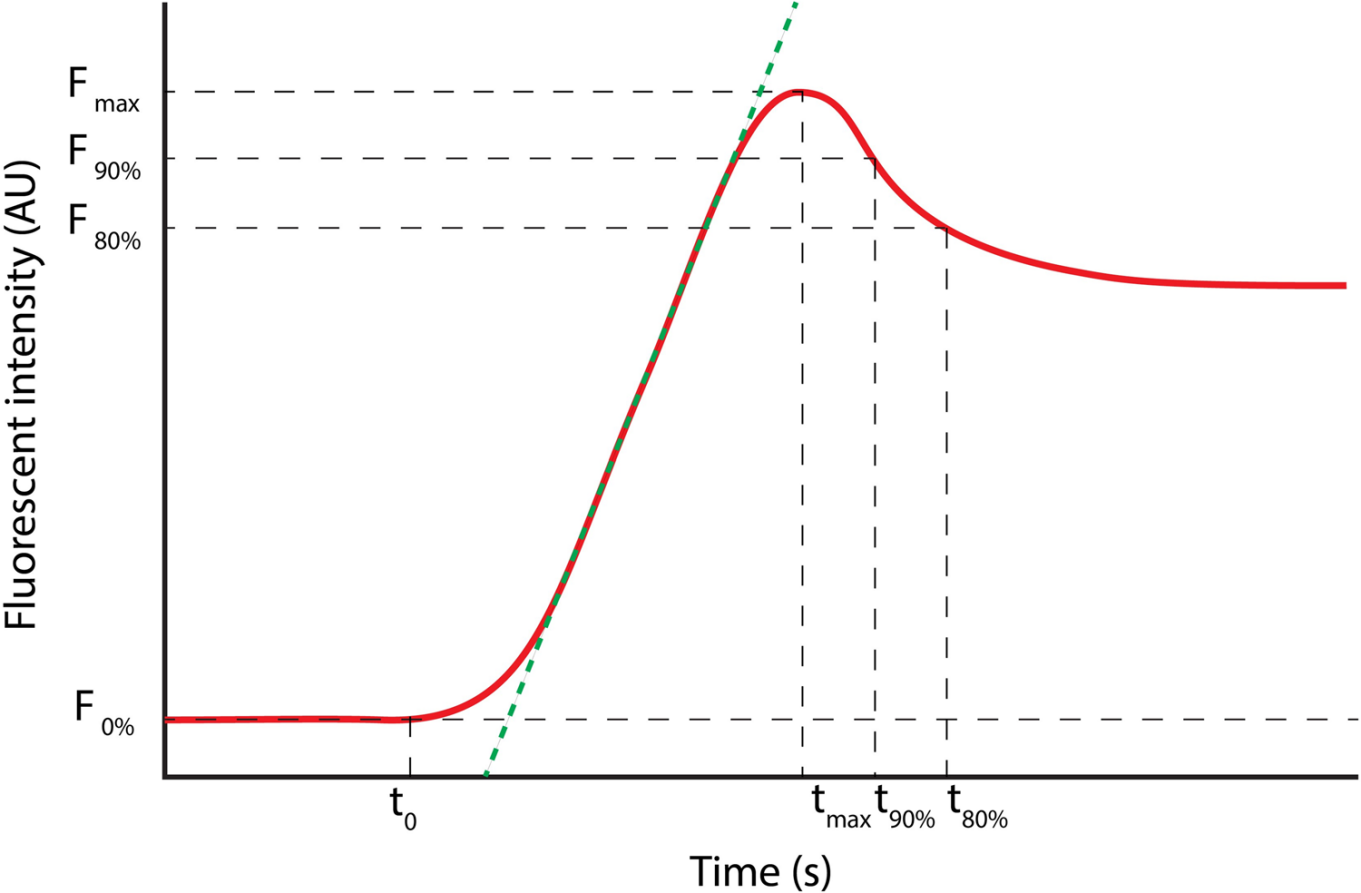
Fluorescence parameters. Influx time point (t_0_): the time point at which the fluorescence intensity in the ROI was statistically significantly larger than the background, F_max_: maximal intensity in arbitrary units (AU), T_max_: time in at which the background-corrected fluorescence intensity reached F_max_, time to peak (ttp): T_max_- t_0_, the green line represents the mean slope: rate at which the fluorescence intensity increased (AU/s), T_90%_: time in seconds until 90% of F_max_ has been reached, T_80%_: time in seconds until 80% of F_max_ has been reached

In order to achieve objective quantification, the raw FA data were analyzed by tailor-made software written in Python on basis of a gray-scale analysis**.** After loading the video into the software, size was calibrated using a measuring tape which was placed in the frame. Subsequently, a circular region of interests (ROI) with a diameter of 1 cm was placed in the midline of the gastric conduit at the planned anastomotic site. Subsequently, the software extracted the mean intensity within the ROI for every frame and plotted the ICG in- and outflow in a fluorescence time curve. A slightly modified version of the arterial input function reported by Elliott et al. was fitted to the curve to reduce the influence of noise on the calculated parameters [18]. From this fit, the following parameters were extracted (Fig. 1): Influx time point (t0): the time point at which the fluorescence intensity in the ROI was statistically significantly larger than in the background, F_max_: maximal intensity in arbitrary units (AU), T_max_: time in seconds from ICG administration until F_max_ has been reached, and time to peak (ttp): time in seconds from t0 until F_max_ has been reached. Mean slope from t0 until F_max_: rate at which the fluorescence intensity increased (AU/s), T_90%_: time in seconds after F_max_ until 90% of F_max_ has been reached, and T_80%_: time in seconds after F_max_ until 80% of F_max_ has been reached (Fig. 2).

### Outcomes and definitions

The primary outcome was the fluorescence parameter the mean slope in relation to anastomotic leakage. Secondary outcomes included other fluorescence parameters, hemodynamic parameters during ICG-FA, AL within 90 days, reinterventions due to AL, 90-day mortality, and change of management due to the ICG-FA assessment. Postoperative anastomotic complications included AL, graft necrosis, and anastomotic stricture. Anastomotic leakage was recorded when an anastomotic defect was objectified by CT scan, during endoscopy or during reoperation, after a clinical or biochemical suspicion (CRP was measured on postoperative day 2 and 3). AL and graft necrosis were defined according to the Esophagectomy Complications Consensus Group classification [19], and complications were classified according to the Clavien–Dindo (CD) score [20]. Clinically relevant benign strictures were defined as a score for dysphagia ≥ 2 and treatment by ≥ 1 dilatation.

### Sample size calculation

In a pilot study in 22 patients undergoing esophagectomy with gastric conduit reconstruction, the mean slope of ICG-FA was quantitatively measured. In the group without AL (*n* = 18), the mean slope was 2.0 (± 2.41) compared to 0.2 (± 0.07) in the group with AL (*n* = 4). [21]. To find a statistical difference in the slope at a significance level of 0.05 and with a power of 80%, the least count of the group with AL should be at least 17 patients.

In a one-year period since the introduction of FA in the Amsterdam UMC, location AMC, the anastomotic leak rate was 14%. To achieve inclusion of at least 17 patients with a leak, in total 122 patients should be included, taking a possible dropout due to technical failures, as well of 15%, at least 135 patients should be included.

### Statistics

Patient characteristics are summarized using descriptive statistics. Categorical data are presented as number of cases and percentages, while continuous data are shown as either mean ± standard deviation or as median and interquartile range (IQR), depending on the data distribution. Fluorescence parameters are reported in median (IQR) and were compared between patients with or without anastomotic complications using the Mann–Whitney U test. A *P*-value < 0.05 was considered statistically significant.

Univariate logistic regression was performed to define a predictive value for fluorescence parameters for AL. When fluorescence parameters had a *P*-value < 0.2, a receiver operating characteristic (ROC) curve was generated [22]. When the ROC curve yielded an area under the curve (AUC) above 0.7, a cut-off value was produced with high specificity and positive predictive value. Specificity was calculated using the Youden’s statistics, after which the positive predictive value was calculated for every specificity.

Data were analyzed using the Statistical Package for Social Sciences (SPSS) of IBM Statistics, version 26.0.

## Results

### Baseline and operative characteristics

**Fig. 3:**
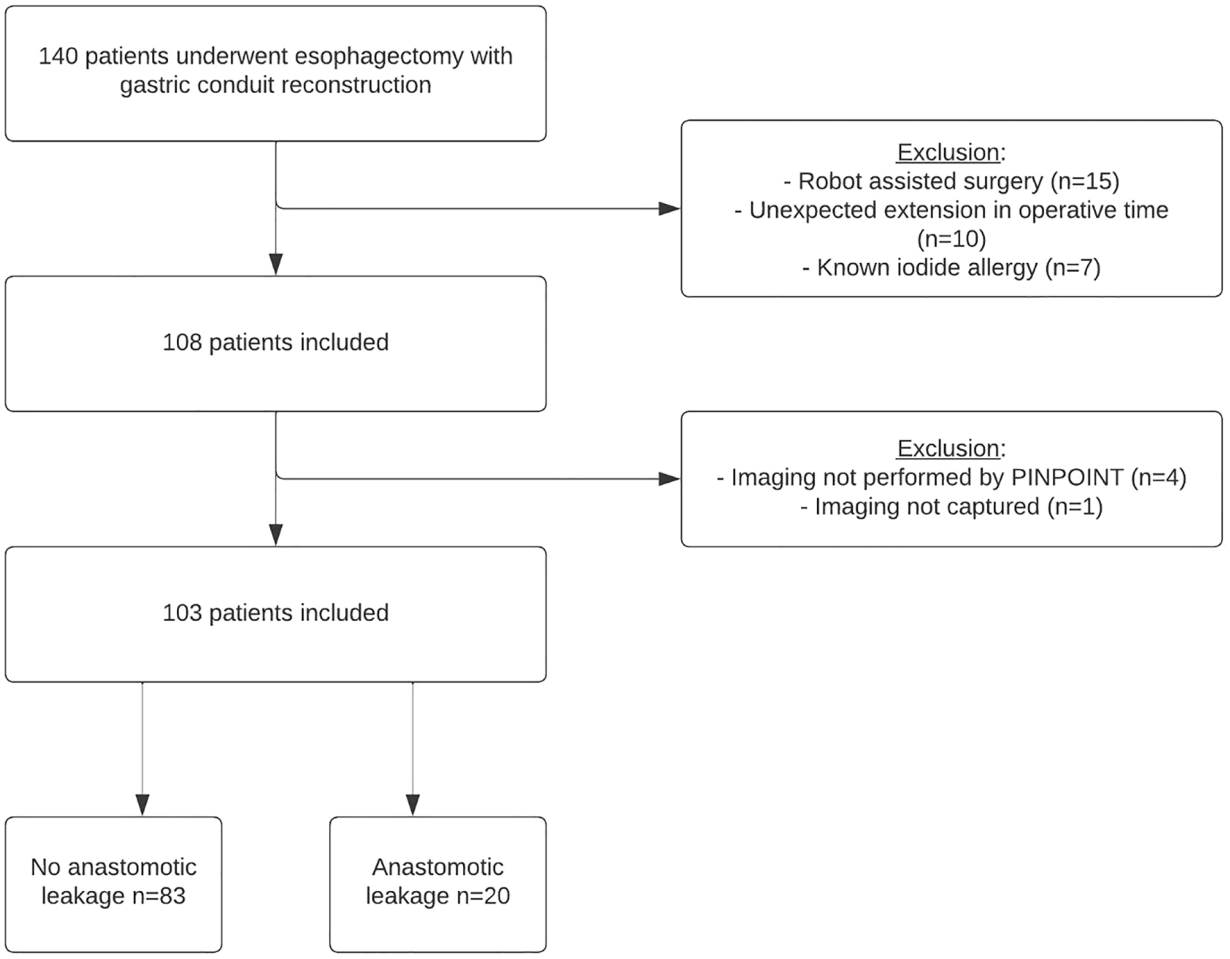
Flowchart of included patients

One hundred forty patients underwent esophagectomy with primary gastric conduit reconstruction from August 2020 to February 2022. One hundred and eight of these patients underwent ICG-FA during surgery and 103 were included in this analysis (Fig. 3). Baseline and surgical details are shown in Table 1. The mean age was 66 ± 9.9 years. The majority of patients were male (79%), received neoadjuvant chemoradiation (85%), and had an adenocarcinoma (81%). The surgical procedure was an Ivor Lewis procedure in 88% of the patients. All procedures were performed (partially) minimally invasively and no conversions were required.

**Table 1:** Baseline and operative characteristics

	All patients (*n* = 103)	No AL (*n* = 83)	AL (*n* = 20)	*p*-value
Age (years) *mean* ± *SD*	65.7 + 9.9	65.2 + 10.5	67.8 + 7.0	0.53
Gender, male	81 (77)	65 (78)	16 (80)	0.57
BMI (kg/m2) *mean* ± *SD*	26.3 + 4.0	26.1 + 3.8	26.9 + 4.7	0.58
Weight loss (kg) at clinical presentation	1 (0–5)	1 (0–5)	1 (0–5)	0.98
ASA ≥ 3	33 (32)	25 (28)	8 (40)	0.28
Smoker, *active*	19 (19)	15 (18)	4 (21)	0.75
Comorbidity				
Pulmonary	11 (10)	10 (12)	1 (5)	0.33
Cardiac	31 (30)	22 (27)	9 (45)	0.09
Diabetes Mellitus	17 (17)	11 (13)	6 (30)	0.08
Tumor histology				
Adenocarcinoma	81 (79)	68 (82)	13 (65)	
Squamous cell carcinoma	15 (15)	9 (11)	6 (30)	
Gastric tumor: adenocarcinoma	6 (6)	5 (6)	1 (5)	
Neuro-endocrine tumor	1 (1)	1 (1)	0	0.18
Immunosuppressant use				
Steroids	5 (5)	3 (4)	2 (10)	
Immunosuppressants	4 (4)	4 (5)	0	0.47
Tumor stage				
cT3	89 (86)	72 (87)	17 (85)	
cN +	56 (54)	43 (52)	13 (65)	0.84
Neoadjuvant Treatment				
Chemoradiation CROSS	87 (84)	71 (86)	19 (95)	
Definitive chemoradiation	3 (3)	3 (4)	0	
Chemotherapy, FLOT	4 (4)	4 (5)	0	
None	9 (9)	8 (10)	1 (5)	0.77
Surgical procedure				
Ivor Lewis	91 (88)	77 (85)	14 (15)	
McKeown	12 (12)	6 (50)	6 (50)	0.15
Approach				
Minimally invasive abdominal	4 (4)	4 (100)	0	
Minimally invasive thorax	6 (6)	5 (83)	1 (17)	
Minimally invasive abdominal and thorax	93 (90)	74 (80)	19 (20)	0.59
Conversion	0	0	0	
Intraoperative complications*	1	0	1	0.81
Estimated bloodloss	200 (100–450)	200 (100–475)	300 (150–438)	0.24
Operative time (min)* mean* ± *SD*	412 ± 71	403 ± 72	454 ± 55	0.05

### Postoperative outcomes

Anastomotic leakage occurred in 20 out of 103 patients (19%). AL rates were 14 out of 91 patients (15%) for intrathoracic and 6 out of 12 (50%) for cervical anastomoses. One patient in the change of management group had AL (*n* = 1/3, 33%). For 11 out of 20 patients (55%) with AL, the CD score was > 3. Reoperation was required for 5 out of 20 patients (25%), including creation of a new anastomosis in two patients, resection of the gastric conduit with an esophagostomy in the neck in two patients, and a video-assisted thoracoscopic surgery to decorticate the lung due to empyema in one patient. An anastomotic stricture occurred in 11 out of 103 patients (11%), of whom none had AL.

Fluorescence parameters for AL patients were also calculated for ROIs 2 cm more proximally on the gastric conduit to compare perfusion if extra mobilization is feasible (Table 2). All parameters improved by changing the anastomotic site into a location more proximal on the gastric conduit: T_max_ 55 (IQR 39–100) versus 65 (44–121) seconds and mean slope 1.0 (IQR 0.3–2.5) and 2.3 (IQR 0.5–3.5).

**Table 2:** Fluorescent parameters at anastomotic site of AL patients

	t_0_	F_max_	T_max_	mean slope
Anastomotic site	25 (19–28)	56 (34–80)	65 (44–121)	1.0 (0.3–2.5)
2 cm more proximal	22 (14–29)	67 (43–84)	55 (39–100)	2.3 (0.5–3.5)

### ICG- FA

Overall, for the pre-anastomotic assessment, overall inflow parameters after ICG injection were 22 s (IQR) for t0 and 51 s (IQR) for Fmax. The mean slope was 1.7 (0.8–3.0) for all patients. In terms of outflow, a median of 15 s (IQR 10–36) until reaching 90% and 34 s until reaching 80% of Fmax (IQR 18–79) were found. Fluorescent parameters were not correlated to cardiac output, heart rate, noradrenaline use or noradrenaline dosage (*P* > 0.05).

An overview of fluorescence parameters in patients with and without AL is shown in Table 3. The mean slope tended to be less steep during both pre-anastomotic as post-anastomotic assessment for the AL group without reaching statistical significance (median of 1.0 versus 1.7, *p* = 0.11 and 1.3 versus 1.0 *p* = 0.76).

**Table 3:** Fluorescent parameters

	Pre-anastomotic assessment			Post-anastomotic assessment		
Parameter	No AL	AL	*p* Value	No AL	AL	*p* Value
T_0_ (sec)	21 (14–26)	25 (19–28)	0.24	16 (11–23)	24 (11–30)	0.09
T_tp_ (sec)	26 (19–40)	39 (25–97)	0.04	32 (23–50)	34 (21–50)	0.84
T_max_ (sec)	51 (37–67)	65 (44–122)	0.03	50 (36–79)	58 (46–81)	0.47
F_max_ (AU)	60 (48–74)	56 (34–80)	0.56	71 (52–86)	62 (56–80)	0.47
Slope(AU/sec)	1.7 (1.0–3.0)	1.0 (0.3–2.5)	0.11	1.7 (0.7–2.2)	1.3 (0.7–1.7)	0.76
T_90%_ (sec)	15 (9–30)	30 (12–41)	0.21	21 (10–42)	33 (15–72)	0.17
T_80%_ (sec)	33 (17–65)	82 (22–111)	0.20	61 (18–91)	71 (49–103)	0.44

Both time values ttp and T_max_ were longer during pre-anastomotic assessment for the AL group in comparison to the non-AL group (39 s vs. 26 s, *p* = 0.04 and 65 vs. 51 s, *p* = 0.03, respectively).

Although not significant, outflow time values during pre both assessments were longer for the AL group compared to the non-AL group (T_80%_ 82 vs. 33 s *p* = 0.21 during pre-anastomotic assessment and 71 versus 61 s, *p* = 0.44 during post-anastomotic assessment). Correspondingly, time values until inflow (t0) of fluorescence during the post-anastomotic assessment tended to be longer too without reaching statistical significance (24 versus 16 s, *p* = 0.09).

Based on subjective ICG-FA interpretation, the surgical team opted for a change of anastomotic site to a clearer fluorescent region 2–5 cm more proximal in the conduit in three out of 103 (3%) patients, requiring extra mobilization of the gastric conduit. One patient (1/3) had anastomotic leakage, diagnosed 10 days postoperatively, complicated by a broncho-esophageal fistula. This was treated by thoracotomy with bronchus repair using a bovine pericardium patch and an intercostal muscle repair and VAC- treatment after reanastomosis, after which the patient recovered well. None of these patients had an anastomotic stenosis.

Univariate analysis was carried out for fluorescence parameters as shown in Table 4, A cut-off value of 97 s for T_max_ was found for anastomotic leakage (*P* = 0.10, AUC = 0.71) with a specificity of 92%.

**Table 4:** Univariate cox proportional hazards regression analysis of anastomotic leakage

Variables	HR (95% CI)	*P* value
Pre-anastomotic t_tp_	1.81 (0.66–5.00)	0.25
Pre-anastomotic T_max_	2.39 (0.84–6.82)	0.10
Pre-anastomotic slope	0.84 (0.32–2.24)	0.73

## Discussion

This study prospectively investigated quantification of ICG-FA by analyzing fluorescence time curves and how it relates to occurrence of AL in patients undergoing esophagectomy with gastric conduit reconstruction. A difference was observed in terms of time until reaching maximal intensity (T_max_) in patients with and without AL during pre-anastomotic assessment. This could be an important parameter which can influence intraoperative decision-making by predicting anastomotic leakage after esophagectomy with gastric conduit reconstruction.

To our knowledge this is the first prospective cohort study to evaluate perfusion with fluorescence time curves in this patient group in a standardized setup. Nevertheless, the phenomenon of quantifying the fluorescence signal in patients undergoing esophagectomy is not new. Measuring time to fluorescence enhancement has already been described as an effective ‘timing’ fluorescence parameter with thresholds established around 90 s until fluorescent enhancement [13, 23]. Absence of fluorescence may suggest arterial insufficiency, whereas a delay in fluorescence in- or outflow may signify venous congestion. These conditions may cause ischemia. However, it is known that when only using subjective visual interpretation of ICG-FA, surgeons overestimate the perfusion compared with quantitative analysis [24]. The slope of the curve is described as having the best clinical performance in identifying AL patients [25, 26]. This study was therefore powered on the mean slope of the curve; however, this did not reach statistical significance.

Research on ICG-FA quantification tends to focus mainly on inflow parameters while defining outflow parameters remains challenging. Moreover, the inflow and outflow of the gastric conduit could affect one and another. For example, severe venous congestion of the gastric conduit can cause reduction of inflow. Our results may emphasize this by longer-time values observed during post-anastomotic assessment until the inflow of ICG (t0). This might explain the less profound differences seen during the post-anastomotic assessment between the AL and non-AL group. Taking this into consideration, it is difficult to distinguish between in- and outflow and it is of paramount importance to not only focus on inflow but also on outflow parameters.

**Fig. 4:**
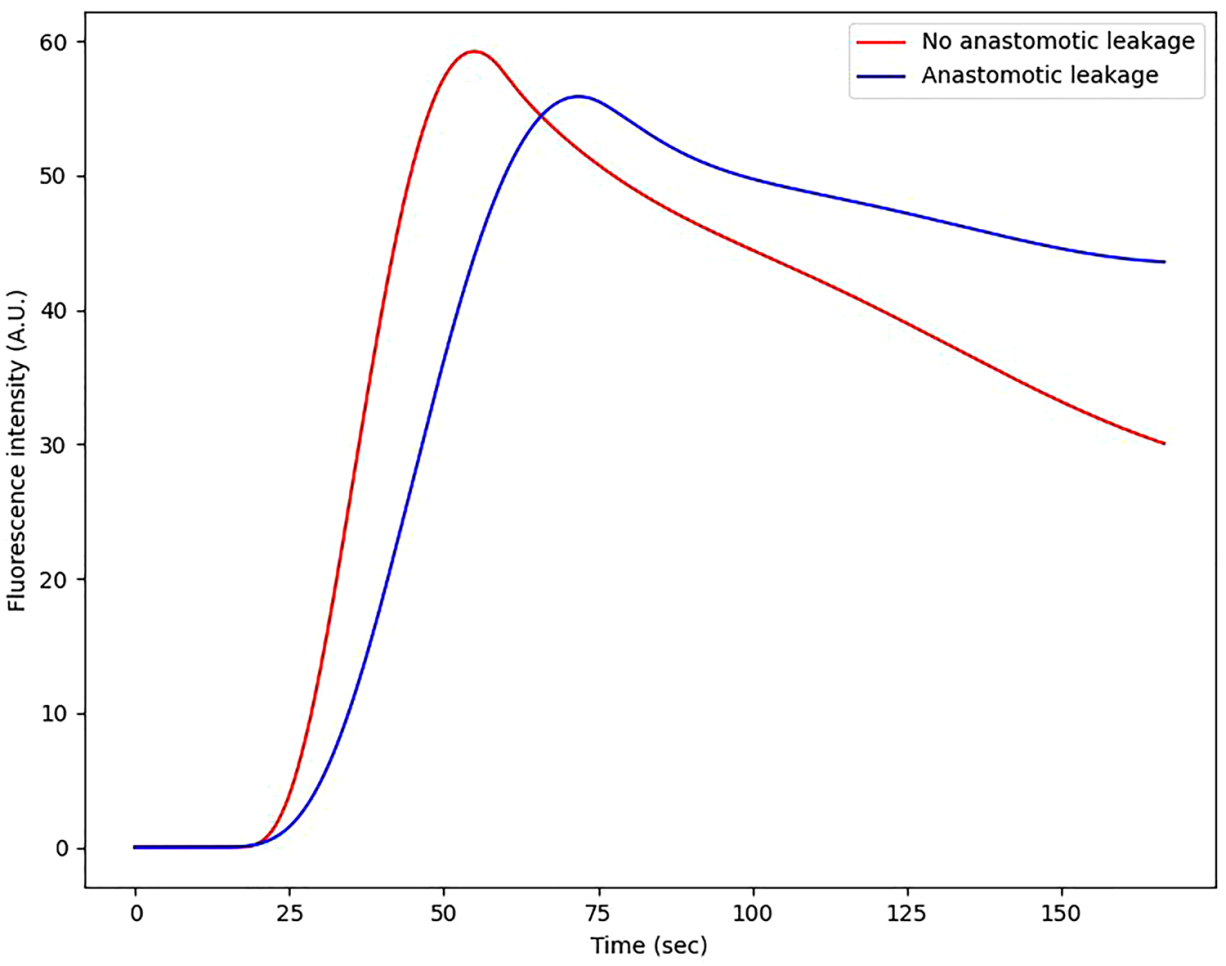
Fluorescence curves of patients with and without anastomotic leakage

Objective fluorescence interpretation by quantitative parameters can help the surgeon intraoperatively; in the patients with AL the perfusion parameters were better 2 cm more proximally; if deemed possible the surgeon could mobilize the gastric conduit more in order to make the anastomosis more proximal. Nevertheless, we know that patients with a change of management (i.e., trimming the gastric conduit) also have high percentages of AL, also in this series, potentially explained by tension on the anastomosis and generally a less perfused gastric conduit [27]. For this reason, ICG-FA might be of more value for determining the postoperative policies than intraoperatively during esophagectomy. Real-time fluorescence time curves are now on the rise in the operating room. The characteristics of the curve in patients with an anastomotic leakage are different from those of patients without anastomotic leakage (Fig. 4). In this way, in future, the surgeon can intraoperatively determine, based on the shape of the curve or validated thresholds, whether a patient has a higher risk of developing an anastomotic leakage. These patients may be selected either for strict postoperative monitoring: early endoscopy or delayed start of enteral feeding or intraoperatively with preemptive endoluminal vacuum-assisted therapy in the form of a VACstent or endosponge placement [28]. On the other hand, in low-risk patients with good perfusion, oral nutrition may be initiated on short notice. In this way, perfusion assessment can be used to better tailor the postoperative course. This approach could reduce length of hospitalization and resources needed to treat complications.

Nevertheless this study is also limited, even with the use of ICG-FA, the AL rate was high, especially in patients undergoing McKeown procedures, which might be explained by the selection of patients for a McKeown procedure at our unit, as these were all patients extended radiation fields to the thoracic inlet/paratracheal region because of higher tumor locations / lymph node metastases located higher up in the mediastinum [29]. This may also be explained by a longer length of the gastric conduit.

As there is a clear difference in leakage incidence as well in the literature as in this study, it might be interesting to look at the fluorescent parameters and curves for the procedures separately. The present study was however not powered to do so. As occurrence of anastomotic leakage involves many different factors not corrected for in this study, a larger prospective trial focusing on interpretation by quantitative threshold focused only on one procedure (for instance Ivor Lewis) with also taking the multifactorial etiology of a leakage would be the next step in validating a threshold. Due to this multifactorial etiology, management determined by ICG-FA could never account for all anastomotic leakages as ICG-FA might only prevent leakages originating from inadequate perfusion. A combined strategy that deals with multiple peri- and intraoperative risk factors of anastomotic leakage might further lower its occurrence.

In this study, only one fluorescence imaging device was used, it is not known how these results relate to other imaging devices as camera settings differ between different manufacturers. Light distribution may differ and this impacts fluorescence intensity in the same field of view. It is of importance to address these aspects in future studies on perfusion assessment using ICG fluorescence imaging to achieve reliable quantification for all systems. In future, fluorescence time curves should be compared between multiple imaging systems and software programs in a standardized setting, for instance, using a phantom. Artificial intelligence could help with the prediction of patient outcomes, while combining FA videos, these imaging characteristics, and patient data [30]. The strength of this study lies in the fact that ICG-FA was performed prospectively in a standardized manner, making it unique in its reproducibility.

In conclusion, quantification of ICG- FA is feasible. Patients with a longer interval from ICG administration until reaching maximum intensity may have an increased risk of anastomotic leakage enabling the early identification of high-risk patients for anastomotic leakage in whom extra mobilization of the gastric conduit or postoperative preemptive measures may be taken. In this fashion ICG-FA might tailor the intra- and postoperative course of patients undergoing esophagectomy with gastric conduit reconstruction. Objective interpretation of ICG-FA should be applied in conjunction with calibration of imaging devices in order to achieve reliable quantification and implement it broadly. A prospective trial focusing on interpretation by quantitative threshold with also taking the multifactorial etiology of anastomotic leakage would be the next step in validating this threshold.
